# Successful termination of ventricular tachycardia with intrinsic anti-tachycardia pacing

**DOI:** 10.1016/j.ipej.2021.06.004

**Published:** 2021-06-22

**Authors:** Itsuro Morishima, Yasunori Kanzaki, Yasuhiro Morita, Yoshinori Tsuji

**Affiliations:** a Department of Cardiology, Ogaki Municipal Hospital, Ogaki, Japan; b Department of Clinical Engineering, Ogaki Municipal Hospital, Ogaki, Japan

**Keywords:** Implantable cardioverter-defibrillator, Intrinsic anti-tachycardia pacing, Ventricular tachycardia

## Abstract

Intrinsic anti-tachycardia pacing (iATP) is a novel automated ATP algorithm that employs post-pacing interval (PPI) to design the next ATP sequence based on an analysis of the prior failed ATP sequence. A patient with hypertrophic cardiomyopathy received an implantable cardioverter-defibrillator (ICD) (Cobalt™ XT DR, Medtronic, Minneapolis, MN, USA) following an episode of syncope due to macro-reentrant ventricular tachycardia (VT) (right bundle branch block configuration, cycle length [CL] 280 ms). The VF zone was set to VTCL <300 ms and iATP therapy was prescribed before and during capacitor charging. The iATP was initiated when VT recurred 3 months later. The first attempt with an assumption of 150 ms propagation time from the pacing site to the VT circuit (9 pulses) could not reset the VT, leaving a PPI of 650 ms. A subsequent attempt involving 20 pulses with an assumption of 250 ms propagation time terminated the VT. Failure to reach the circuit is a major cause of unsuccessful ATP. In this regard, iATP is expected to have theoretical advantages over empirical and traditional ATP therapies. To the best of our knowledge, this is the first intracardiac electrogram illustrating how automated precision ATP terminates VT in a clinical setting.

Intrinsic anti-tachycardia pacing (iATP) is a novel automated ATP algorithm that employs post-pacing interval (PPI) to design the next ATP sequence based on analysis of the prior failed ATP sequence [1,2]. A male patient with hypertrophic cardiomyopathy received an implantable cardioverter-defibrillator (ICD) (Cobalt™ XT DR, Medtronic, Minneapolis, MN, USA) following an episode of syncope due to macro-reentrant monomorphic ventricular tachycardia (VT) (right bundle branch block configuration, inferior axis, cycle length [CL] 280 ms). The VF zone was set to a VTCL <300 ms, and iATP therapy was prescribed before and during capacitor charging. The first ATP was initiated when VT recurred three months later (VTCL 280 ms). The sequence included eight S1 pulses with a coupling interval (CI) of 88% of the VTCL (240 ms) followed by an S2 pulse with a CI of 220 ms (Fig. 1). The number of S1 pulses was automatically determined to reach a hypothetical VT circuit with a default assumption of 150 ms propagation time (Fig. 2A) [1]. However, the VT was not terminated, and the PPI of 650 ms was greater than the maximum reset zone, which was defined as propagation time x 2 plus VTCL (150 × 2 + 280 = 580 ms), indicating failure of the ATP sequence to reset the VT (Fig. 1, Fig. 2B). Subsequently, the number of S1 pulses increased to 19 with an assumption of 250 ms propagation time from the pacing site to the VT circuit in the second ATP sequence. The VT was terminated, and shock was aborted (Fig. 1).

**Fig. 1:**
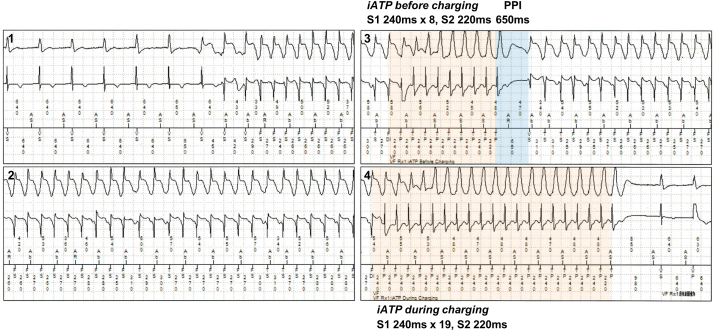
Stored electrocardiogram of the event leading to ventricular tachycardia termination by the second intrinsic anti-tachycardia pacing (iATP) sequence.

**Fig. 2:**
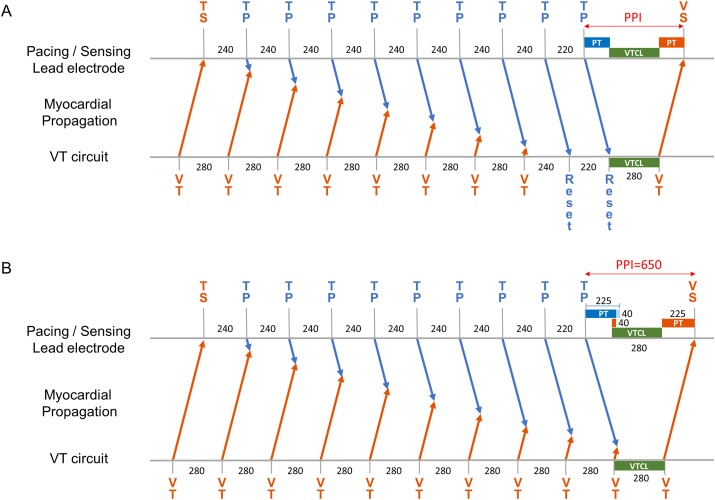
Resetting (A) and no resetting (B) of ventricular tachycardia (VT) by the initial iATP sequence (TP). **A.** If the myocardial propagation time (PT) were within a default assumption of 150 ms propagation time, the VT resetting would be observed with nine ATP pulses, and the post pacing interval (PPI) would be within PT x 2 + VT cycle length (CL) (=580 ms). **B.** The long PPI indicates no resetting of the VT and the myocardial PT longer than 150 ms. Suppose the myocardial PT is 225 ms and the final iATP pulse collides with the VT wavefront 40 ms outside the VT circuit, the PPI would theoretically turn out to be 650 ms. TS, sensed tachycardia; VS, sensed V wave.

Failure to reach the circuit is one of the major causes of unsuccessful ATP [2]. In this regard, iATP is expected to have theoretical advantages over empirical and traditional ATP therapies [2]. The first failed ATP sequence is extremely similar to the commonly used empirical ATP; however, the second successful sequence with eleven additional S1 pulses (with the same pacing CL) appears unusual in traditional practice. To the best of our knowledge, this is the first intracardiac electrogram illustrating how automated precision ATP works to terminate VT in a clinical setting.

## Conflicts of interest

None.

## Funding

None.

## Declaration of comepting interest

The authors declare that they have no known competing financial interests or personal relationships that could have appeared to influence the work reported in this paper.
